# An artificial HSE promoter for efficient and selective detection of heat shock pathway activity

**DOI:** 10.1007/s12192-014-0540-5

**Published:** 2014-08-29

**Authors:** Viktoria Ortner, Alfred Ludwig, Elisabeth Riegel, Sarah Dunzinger, Thomas Czerny

**Affiliations:** 1 Department of Applied Life Sciences, University of Applied Sciences, FH Campus Wien, Helmut-Qualtinger-Gasse 2, A-1030, Vienna, Austria; 2 Department of Agrarian Production, Genetics and Microbiology Research Group Public, University of Navarre, Pamplona, Navarre Spain

**Keywords:** Heat shock, HSF1, Reporter, Burn

## Abstract

Detection of cellular stress is of major importance for the survival of cells. During evolution, a network of stress pathways developed, with the heat shock (HS) response playing a major role. The key transcription factor mediating HS signalling activity in mammalian cells is the HS factor HSF1. When activated it binds to the heat shock elements (HSE) in the promoters of target genes like heat shock protein (HSP) genes. They are induced by HSF1 but in addition they integrate multiple signals from different stress pathways. Here, we developed an artificial promoter consisting only of HSEs and therefore selectively reacting to HSF-mediated pathway activation. The promoter is highly inducible but has an extreme low basal level. Direct comparison with the HSPA1A promoter activity indicates that heat-dependent expression can be fully recapitulated by isolated HSEs in human cells. Using this sensitive reporter, we measured the HS response for different temperatures and exposure times. In particular, long heat induction times of 1 or 2 h were compared with short heat durations down to 1 min, conditions typical for burn injuries. We found similar responses to both long and short heat durations but at completely different temperatures. Exposure times of 2 h result in pathway activation at 41 to 44 °C, whereas heat pulses of 1 min lead to a maximum HS response between 47 and 50 °C. The results suggest that the HS response is initiated by a combination of temperature and exposure time but not by a certain threshold temperature.

**Electronic supplementary material:**

The online version of this article (doi:10.1007/s12192-014-0540-5) contains supplementary material, which is available to authorized users.

## Introduction

The heat shock (HS) response is a highly conserved stress response of all cells from bacteria to humans but with differences in the involved proteins and their regulation. Although the HS pathway was initially discovered as a reaction to higher temperatures, it was later shown that cells use this response when exposed to different kinds of cellular stress (Morimoto 1993). When exposed to heat stress, native proteins in the cells start to partially unfold. In addition, protein expression is strongly affected by inhibition of RNA splicing (Yost and Lindquist 1986) and downregulation of translation initiation (Spriggs et al. 2010). Beside the intracellular effects, stress also acts on the cell membrane initialising hyperfluidisation and rearrangement of microdomains (Török et al. 2013). To prevent cells from further destruction, the HS signalling pathway is induced (Morimoto 1993).

The HS response can be activated by both external and internal triggers, leading to the activation of the transcription factor HSF1, which then turns on expression of stress-responsive genes. HSF1 is activated by a multi-step process converting the inactive monomer into a transcriptionally active trimeric version. The monomeric state is maintained by formation of a chaperone complex including HSP90 (Ali et al. 1998). In addition, trimerisation is inhibited by intramolecular interaction of N- and C-terminal domains of HSF1 (Rabindran et al. 1991). Furthermore, the transactivation capacity of HSF1 is inhibited by binding of the chaperone HSP70 together with its co-chaperone HSP40 (Shi et al. 1998). In response to stress stimuli, HSF1 monomers are released from the complex and trimerise either as homotrimers or as heterotrimers containing HSF1 and HSF2 (Ostling et al. 2007; Vihervaara et al. 2013). The trimers become localised to the nucleus by blocking nuclear export (Mercier et al. 1999; Vujanac et al. 2005) and bind to target gene promoters.

The active HSF trimers induce the expression of several ‘survival proteins’, most of them preventing cell death and enhancing survival like the heat shock proteins (HSPs). HSP90 and HSP70 are prominent chaperones necessary for proteostasis, which are recruited to the stress-induced unfolded proteins. As HSP90 and HSP70 are also major components of the HSF inactivation complex, this explains the HSF1 release and transcriptional activation of target genes. Subsequent accumulation of HSPs in the cell stops this process, creating a self-regulatory mechanism in HSP expression (reviewed in Akerfelt et al. 2010). Another level of regulation focuses on HSF1 modification and involves other cellular signalling cascades. Regulation at this level is mediated by posttranslational modification, HSF1 localisation or trimerisation (reviewed in Anckar and Sistonen 2011). The active HSF1 trimer binds to the HSEs in the promoter of target genes as for example HSPA1A (HSP72) and induces transcription. In *Drosophila*, paused RNA polymerase II was found to be associated with the 5′ end of the uninduced *hsp70* gene and released upon heat treatment (O’Brien and Lis 1991). Nucleosomal packaging in combination with RNA polymerase pausing was also subsequently proposed to be the rate-limiting step in mammalian HSP gene expression (Brown et al. 1996), with HSF1 releasing the RNA polymerase.

On contrary to the conditions discussed so far, excessive temperatures have devastating effects on living cells, resulting in membrane disruption and severe denaturation of proteins (Orgill et al. 2005). Cells directly exposed to such temperatures during burn injuries form immediately a necrotic zone of coagulation. Less-affected cells in the zone of stasis activate emergency pathways like the HS response. Unfortunately, they not only suffer the burden of denatured proteins, but in addition face a worsening environment of decreased perfusion, lack of oxygen, and a massive inflammatory response. Consequently, these tissue layers can become necrotic in a process known as burn wound progression. For the quality of life after recovery, the preservation of deep skin layers of the patient is of critical importance (Shupp et al. 2010). So far, little is known about the regulation and the kinetics of the HS response in cells exposed to high temperatures for short time.

Exact measurement of pathway activity is of major importance for the evaluation of its regulation. We, therefore, developed a novel artificial reporter reacting exclusively to HSF-mediated activity. The construct is highly sensitive to heat stress and represents a reliable and selective reporter for HS pathway activity. Using it for recording of pathway activity after different heat treatments, we found similar responses to both extended, as well as short (1 min) exposure times, but at completely different temperatures.

## Materials and methods

### RNA isolation and cDNA synthesis

Total RNA was isolated from HEK293 cells according to the manufactures protocol using Invisorb® Spin Tissue RNA Mini Kit (Invitec). Residual DNA was removed with DNAse I (Thermo) according to the manufacturer’s instructions, and RNA was transcribed into complementary DNA (cDNA) using random hexamer primers (100 μM, Thermo) and RevertAidTM H Minus M-MuLV Reverse Transcriptase (Thermo) according to the manufacturer’s protocol.

### qPCR

For quantitative polymerase chain reaction (qPCR), Taqman probes were designed using Primer Express V2 and cDNA was analysed in an Mx3000P (Strategene) qPCR cycler. As an endogenous control, glyceraldehyde-3-phosphate dehydrogenase (GAPDH) was used. hGAPDH (NM_001256799.1) forward: 5′-GGAAGGTGAAGGTCGGAGTCAA-3′, reverse: 5′-ACCAGAGTTAAAAGCAGCCCTG-3′, probe: 5′-HEX-ATTTGGTCGTATTGGGCGCCTGGTC-BHQ1-3′, and qPCR settings: buffer B, 3.5 mM MgCl_2_, eff., 97–99 %. Firefly luciferase: forward: 5′-TGGATTACGTCGCCAGTCAAG-3′, reverse: 5′-TTCGGTACTTCGTCCACAAACA-3′, probe: 5′-FAM-CGCGAAAAGTTGCGCGGAGG-BHQ1-3′, and qPCR settings: buffer B, 3.5 mM MgCl_2_, eff., 96–98 %. HSPA1A (NM_005345.5): forward: 5′-AACCAGGTGGCGCTGAAC-3′, reverse: 5′-TGGAAAGGCCAGTGCTTCAT-3′, probe: 5′-FAM-AACACCGTGTTTGACGCGAAGCG-BHQ1-3′, and qPCR settings: buffer A, 3 mM MgCl_2_, eff., 92–95 %. Per reaction, 1.5 μl cDNA, 2 μM primers, 2 μM Taqman hydrolysis probe, 1× buffer B (80 mM Tris, 20 mM (NH_4_)2SO_4_, 0.02 % Tween 20) or 1× buffer A (10 mM Tris, 50 mM KCl), 3–3.5 mM MgCl_2_, 0.2 mM dNTP mix (Thermo), and 0.025U Taq polymerase (Agrobiogen) were adjusted to a final volume of 25 μl with water. Temperature protocol: 5 min (95 °C)—40 cycles of 30 s (95 °C) and 60 s (60 °C). All qPCRs were performed in triplicates and normalised to the GAPDH levels.

### Plasmids

Different numbers of HSEs (CTCGAGAACGTTCTAGAACGTCGAC) with flanking restriction sites were cloned into pMlucF upstream of the Fos minimal promoter driving firefly luciferase. The human HSPA1A (NM_005345.5) reporter construct (pMluc HSP72 promA) contains a HSPA1A promoter fragment (−712 to +1; lacking the 5′ untranslated part of HSPA1A messenger RNA (mRNA)) driving firefly luciferase. Extended constructs for the HSPA1A promoter contain in addition regions −1,615 to–713 (pMluc HSP72 promAB) and +1 to +244 (at position 244, the HSPA1A ATG was directly fused to luciferase; pMluc HSP72 prom AB5′). The fragments were isolated by PCR from human genomic DNA and verified by sequencing. pMC Gluc, containing Gaussia luciferase cDNA of pGLuc Basic (NEB) in the pMC vector (Fink et al. 2006) or pRL-CMV (Promega), containing Renilla luciferase (both under control of the CMV promoter) were used as internal references.

### Cell culture and transfection

HeLa, MCF-7, HaCaT, SK-BR3, mouse embryonic fibroblast (MEF) wildtype and HSF1 (−/−) cells (McMillan et al. 1998) and HEK293T, HEK293 and C5 cells based on HEK293 (Ortner et al. 2012) were grown in Dulbecco’s modified Eagle’s medium (DMEM) high glucose and NIC NIH-383 cells in RPMI 1640. All media were supplemented with 10 % FCS and 1× penicillin/streptomycin (all PAA), and cells were grown at 37 °C in a humidified environment of 5 % CO_2_. For transient transfection experiments, 0.3 × 10^5^ cells were seeded in a 24-well plate, incubated for 24 h at 37 °C and transfected with Turbofect (Thermo) according to the instructions of the manufacturer (400 ng DNA in total). Cells were incubated for an additional 24 h before heat treatment.

### Heat treatment

For transient transfections, 0.3 × 10^5^ cells were seeded into 24 well plates, transfected 2 days later and cultivated for an additional 1 day at 37 °C. For heat treatment, cells were incubated at a temperature of 41–46 °C for the indicated time and then returned to 37 °C.

Heat treatment of the C5 cells in the thermocycler was performed with 10^5^ cells in suspension in 100 μl DMEM complete + 5 mM Hepes (pH 7.4), using a PCR tube. The temperature programme was 3 min at 37 °C, 1–120 min at the indicated temperatures and 5 min at 37 °C. For recovery, the cells were resuspended in 1 ml DMEM complete, transferred to a 1.5-ml reaction tube and returned to 37 °C or directly used for the experiments.

### Luciferase assay

For firefly luciferase activity determination cells were lysed in 50 μl lysis buffer (0.1 M Tris pH 7.5, 1 % Triton X) and luciferase activity measurement was performed in a LUMAT LB 9705 luminometer. For detecting firefly and Gaussia luciferase, 40 μl of the cell lysate was used for firefly luciferase assay (injection of 100 μl substrate solution 6.25 mM Tris pH 7.5, 10 mM MgCl, 2.5 mM ATP, 100 μM Dluciferin) and 10 μl of the cell lysate was used for Gaussia luciferase assay (injection of 100 μl substrate solution 2.5 mM EDTA, 6.25 mM Tris pH 7.5, 3 μM coelentrazine). For normalisation, the firefly luciferase RLU values were divided by those for Gaussia luciferase.

### EMSA and cellular extracts

HEK293 cells were treated for 60 min at 43 °C in a 10-cm cell culture dish and then kept for 1 h at 37 °C. Nuclear extracts were prepared from 1 × 10^6^ cells according to Schreiber et al. (1989). Electrophoretic mobility shift assay (EMSA) was performed as described previously (Czerny et al. 1993); 0.04-pmol-labelled HSF1 probe containing one consensus HSE (Cunniff and Morgan 1993) was added to each reaction: 5′-TCGACCTGGCGAATGGGGCCTGAAGAACGTTCTAGAACTTCCTCTCTGC-3′ and 5′-TCGAGCAGAGAGGAAGTTCTAGAACGTTCTTCAGGCCCCATTCGCCAGG-3′ together with nuclear extract containing 2 μg protein. For competition, 20 pmol of a double-stranded oligonucleotide containing 2 HSEs was added: 5′-TCGAGAACGTTCTAGAACTGGAGAACGTTCTAGAACG-3′ and 5′-TCGACGTTCTAGAACGTTCTCCAGTTCTAGAACGTTC-3′.

## Results

### The artificial HSE promoter detects HSF activity with high sensitivity

**Fig. 1:**
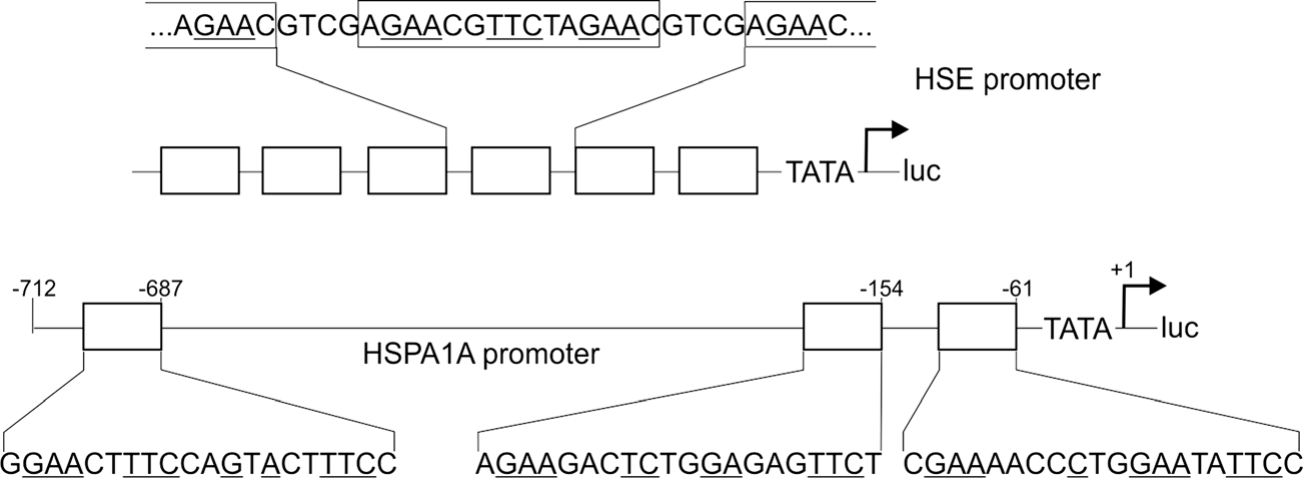
Schematic view of HSE and HSPA1A promoter constructs. A HSE promoter containing six optimised HSEs is compared with the natural HSPA1A promoter ranging from −712 to the transcription start site. Sequences of the HSEs are shown with the core elements of each pentamer *underlined*

Several HSP genes have been identified as transcriptional targets of the HS response and can therefore be used as markers for pathway activity. The prototype of a HS-inducible gene is HSPA1A (HSP72; Wu et al. 1986). It is strongly induced upon different stress conditions and has therefore repeatedly been used for detection of HS pathway activity. However, similar to other target genes, HSPA1A does not exclusively react to the HS response but instead integrates inputs from various stress-dependent and stress-independent pathways (Sasi et al. 2014). As a consequence, the HSPA1A promoter is not ideal for selective detection of HS pathway activity. The binding of trimerised and posttranslationally modified HSF1 to the HSEs of target gene promoters is the key event for signal transduction within the HS pathway (Akerfelt et al. 2010). Hence, an ideal reporter should selectively react to transcriptional activity of this factor. We therefore generated an artificial promoter consisting only of HSEs (Fig. 1).

**Fig. 2:**
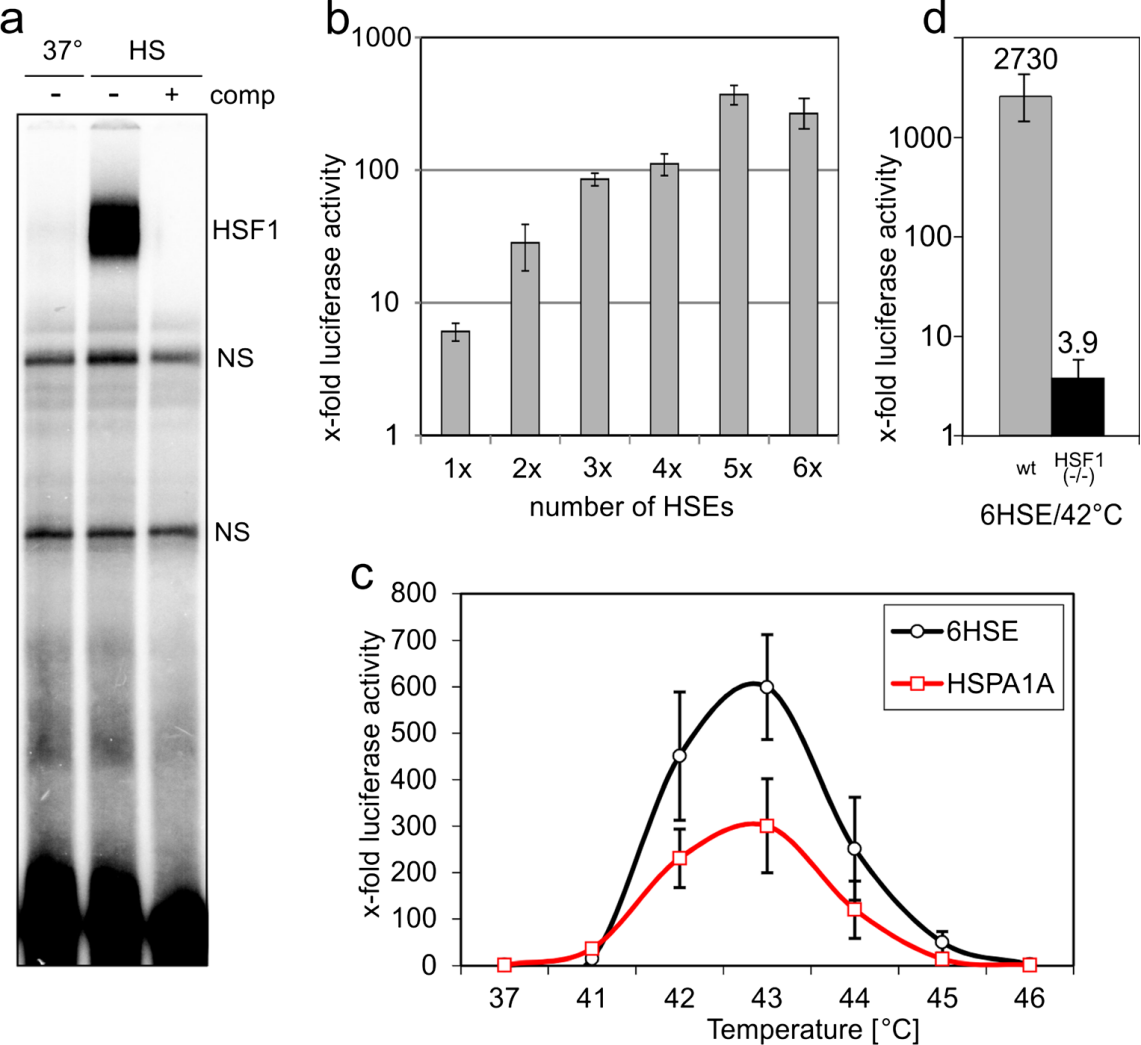
Artificial HSE promoter for sensitive detection of HSF activity. **a** EMSA of nuclear extracts prepared from HEK 293 cells treated at 43 °C for 2 and 1 h of recovery (*HS*) or reference cells kept at 37 °C. To ensure specificity of the detected bands, competitor DNA was added to one reaction at a 500-fold excess (*comp*; note that the oligo used for competition differs from that used as probe and therefore does not affect the nonspecific complexes (*NS*)). **b** MEF wildtype cells were transiently transfected with reporter constructs containing the indicated numbers of HSEs. After 24 h, the cells were heat treated for 2 h at 43 °C and luciferase activity determined after 6 h of recovery. The luciferase values for each construct were normalised to that of cells transfected with the same constructs but kept at 37 °C, in addition a Gaussia luciferase expression construct was used as internal reference. **c** Transient transfection of HEK 293 cells with a 6× HSE containing or a HSPA1A promoter containing reporter. Heat treatment, luciferase activity measurements and normalisation as described in (**b**). **d** Wildtype and HSF1 (−/−) MEF cells were transiently transfected with a 6× HSE promoter construct and treated for 1 h at 42 °C, luciferase activity was measured 6 h later and is shown after normalisation to an internal Gaussia luciferase expression plasmid (relative to levels measured at 37 °C). The experiments in (**b**)–(**d**) were performed in triplicates, and SEM was calculated

The DNA-binding properties of HSF1 have been analysed in detail (Xiao et al. 1991), and a sequence consisting of three inverted GTTCT pentameric elements was identified as an optimised HSE (Cunniff and Morgan 1993). We verified the high affinity of this HSE sequence in an EMSA experiment and observed strong DNA-binding activity in extracts of heat-treated HEK293 cells (43 °C) but not in those of reference cells kept at 37 °C (Fig. 2a). We next tested this HSE in an artificial promoter. In combination with a TATA box, even a single HSE could substantially upregulate luciferase activity in response to heat stress. Compared with cells kept at 37 °C, heat treatment of 60 min at 43 °C resulted in a 6-fold induction of luciferase levels in transiently transfected MEF cells (Fig. 2b). Multimerisation of the HSE led to further activation, with a maximum induction of reporter gene activity of almost 400-fold for five HSE copies (Fig. 2b). Therefore, multimerisation of HSEs results in a synergistic activation of HSF-mediated transcription.

We next compared the artificial HSE promoter with the HSPA1A promoter. We used a 713-bp fragment of the promoter starting from the TATA box (Wu et al. 1986). This promoter fragment contains three HSEs (Fig. 1), two of these are positioned close to the transcription initiation site (Wu et al. 1986; Koizumi et al. 2013) and one almost 700 bp upstream (Trinklein and Murray 2004). Due to the complex architecture of the natural version (Sasi et al. 2014), we expected differences in the stress response of the two promoters. Indeed, the HSPA1A promoter showed a dramatically higher basal level in HEK293 cells at 37 °C, which can be explained by multiple supportive elements of the natural promoter compared with the artificial HSE construct. Both promoters were highly inducible at temperatures between 41 and 44 °C (2 h heat duration), but the induction rates of the HSE promoter were higher compared with that of HSPA1A (Fig. 2c). Despite the differences in the induction levels, the resulting temperature profile between 41 and 44 °C was highly similar, indicating an identical underlying pathway. We extended these experiments to other cell lines in order to exclude cell type specific results (Fig. S1). The temperature profile was surprisingly similar in all cell lines tested. Peak activities appeared at 42 °C, except for keratinocytes (HaCaT), which showed maximum activity at 43 °C. In all cell lines (except for HEK293T), the artificial promoter showed higher induction ratios compared with the HSPA1A promoter. Therefore, the HSE promoter consistently detected HS response activity in all cell lines tested.

The complex HSPA1A promoter showed reactions to heat stress comparable to the simplest version of a HS-responsive promoter containing exclusively HSEs in combination with a TATA box. It could, however, be that the promoter fragment that we used does not recapitulate the full regulatory spectrum of the *HSPA1A* gene. We, therefore, also tested extended versions of the HSPA1A promoter, starting 1.6 kb upstream of the transcription initiation site (this extension is already positioned within the first intron of the neighbouring *HSPA1L* gene), and we also included the 5′ untranslated region of the HSPA1A mRNA (in this case, the luciferase coding region was fused in frame to the AUG of the HSPA1A protein, therefore including potential polymerase pausing sequences). These extended versions of the promoter showed the same activation pattern for the different temperatures, but lower induction levels as the main promoter fragment (data not shown). Therefore, in response to heat stress, isolated HSEs can perfectly mimic the transcriptional activation of a complex natural heat-responsive promoter in transient cell culture experiments.

HSF1 is considered to be the principal regulator of the HS response; however, other HSFs also bind to HSEs (Yamamoto et al. 2009). HSF4 has a specialised function in sensory organs but plays no role in the HS response (Akerfelt et al. 2010); however, HSF2 has been shown to bind HSEs in response to cellular stress (Vihervaara et al. 2013). In order to test the dependence of transcriptional activation of the HSE promoter on HSF1, we performed experiments in HSF1 (−/−) MEF cells (McMillan et al. 1998). Heat exposure resulted in almost 3,000-fold higher luciferase levels in MEF wildtype cells, whereas 3.9-fold activation was measured in HSF1 (−/−) cells (Fig. 2d). The heat exposure of 42 °C did not affect viability of the HSF1 (−/−) cells during the experiment, as could be seen from Renilla luciferase activity measured from a co-transfected plasmid with a constitutively active promoter. Similar experiments with the HSPA1A promoter (data not shown) resulted in 53-fold activation in wildtype and 6.3 in HSF1 (−/−) cells. Comparing the residual promoter activity shows that the HSE promoter was activated to 0.1 % in absence of HSF1 (potential contribution by HSF2), but the HSPA1A promoter to 12 %. This suggests that the *HSPA1A* gene is induced by more than one stress pathway in response to heat treatment, whereas the HSE reporter strongly depends on HSF1 activity.

### Effects of temperature and duration of heat exposure on the HS response

Having established a sensitive tool for detection and quantification of HSF1 activity, we used the HSE promoter to determine pathway activity under different stress conditions. We tested its response to different heat conditions by varying the temperature and the heat duration. In particular, we were interested how extreme short exposure times as occurring in burn injuries affect the pathway. For gene expression studies in response to heat treatment different temperatures in the range of 41–44 °C and treatment durations of 30 min up to 6 h have repeatedly been used in the literature. Reproducible and controlled experiments with shorter exposure times are difficult to perform, due to the slow heat transfer in standard cell culture equipment. We, therefore, incubated cells in suspension in small PCR tubes which allow accurate temperature control in thermocyclers even for short exposure times. For the experiments, we used HEK293 cells harbouring a stably integrated HSE promoter in combination with the reporter gene firefly luciferase (Clone C5; Bajoghli et al. 2004; Ortner et al. 2012). Luciferase activity was measured 6 h after heat treatment. Measurements of the interior temperature in the PCR tubes indicated a delay of 10 to 15 s before the cycler temperature was reached. In order to assure reliable temperature control, we therefore limited the exposure time to 1 min. Applying this experimental setup, we used heat durations of 120, 60, 30, 10, 3 and 1 min and also varied the temperature over a broad range.

**Fig. 3:**
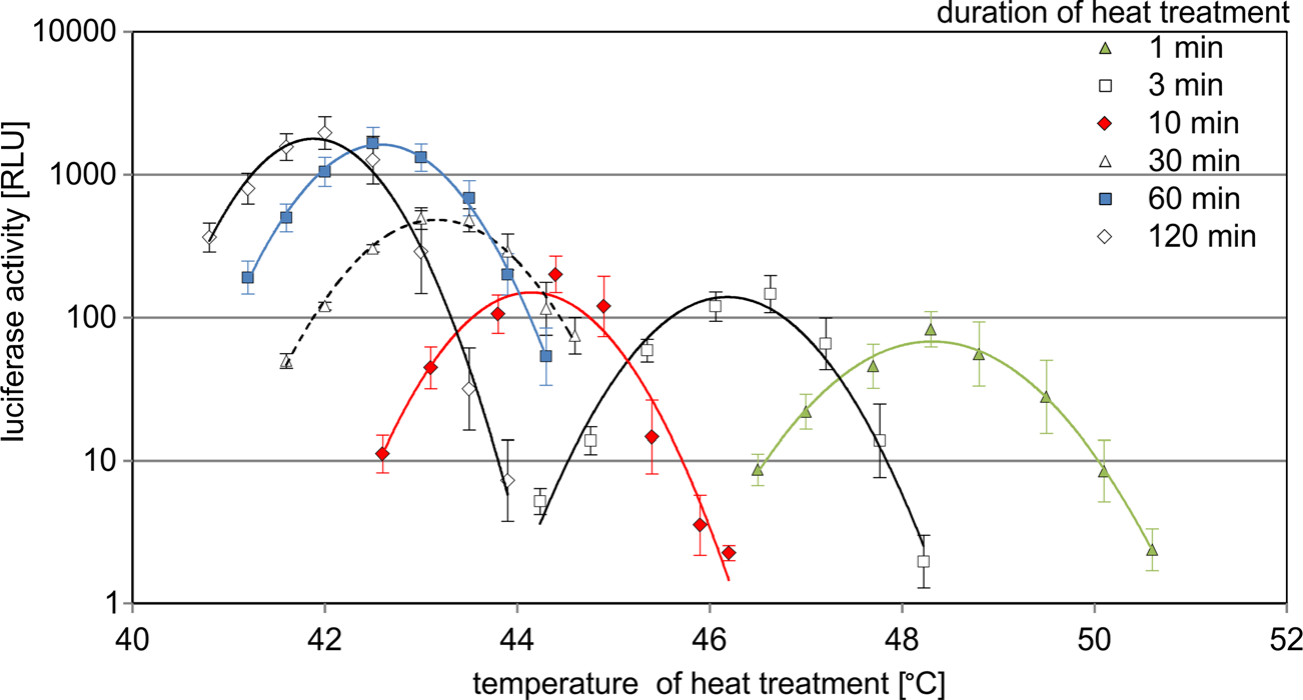
HS response for different heat exposure times. The HSE promoter cell line was incubated at different temperatures for 1–120 min in a thermocycler. Luciferase protein activity was determined 6 h after heat treatment and was normalised to the values for 37 °C reference cells. All experiments were performed in triplicates, and SEM was calculated

**Fig. 4:**
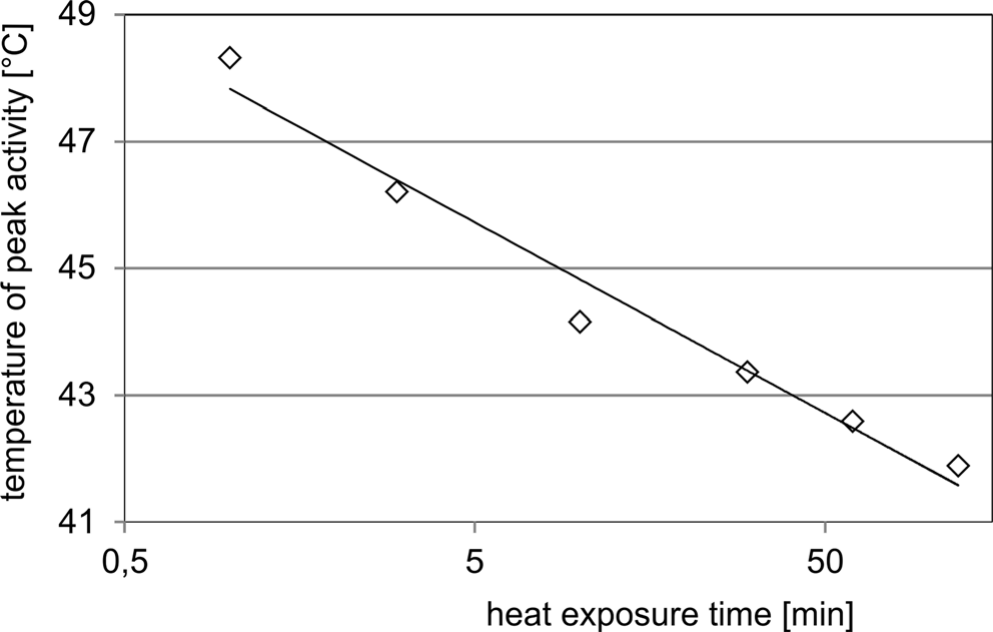
Temperature dependence of HS response. Temperatures of the peak values shown in Fig. 3 are plotted against the heat exposure time

The HSE promoter activity for 1- and 2-h heat exposure was observed in the expected range of 41 to 44 °C (Fig. 3). However, shorter durations resulted in a stepwise shift to higher temperatures. 1 min exposure finally showed pathway activity between 47 and 50 °C (peak activity calculated at 48.3 °C). Interestingly, the pattern of the individual activation curves was quite similar, all were narrow, covering 3 to 4 °C, but centred at different peak temperatures depending on the heat duration. When the peak temperatures are plotted against the heat duration, an almost linear dependence can be detected (logarithmic scale of exposure time; Fig. 4). The maximum induction of the promoter was stronger for 60 and 120 min exposure compared with the peak levels for heat durations below 10 min (Fig. 3). Therefore, HS response activation strictly depends on a combination of temperature and heat duration.

### Influence of heat duration on HS promoter kinetics

**Fig. 5:**
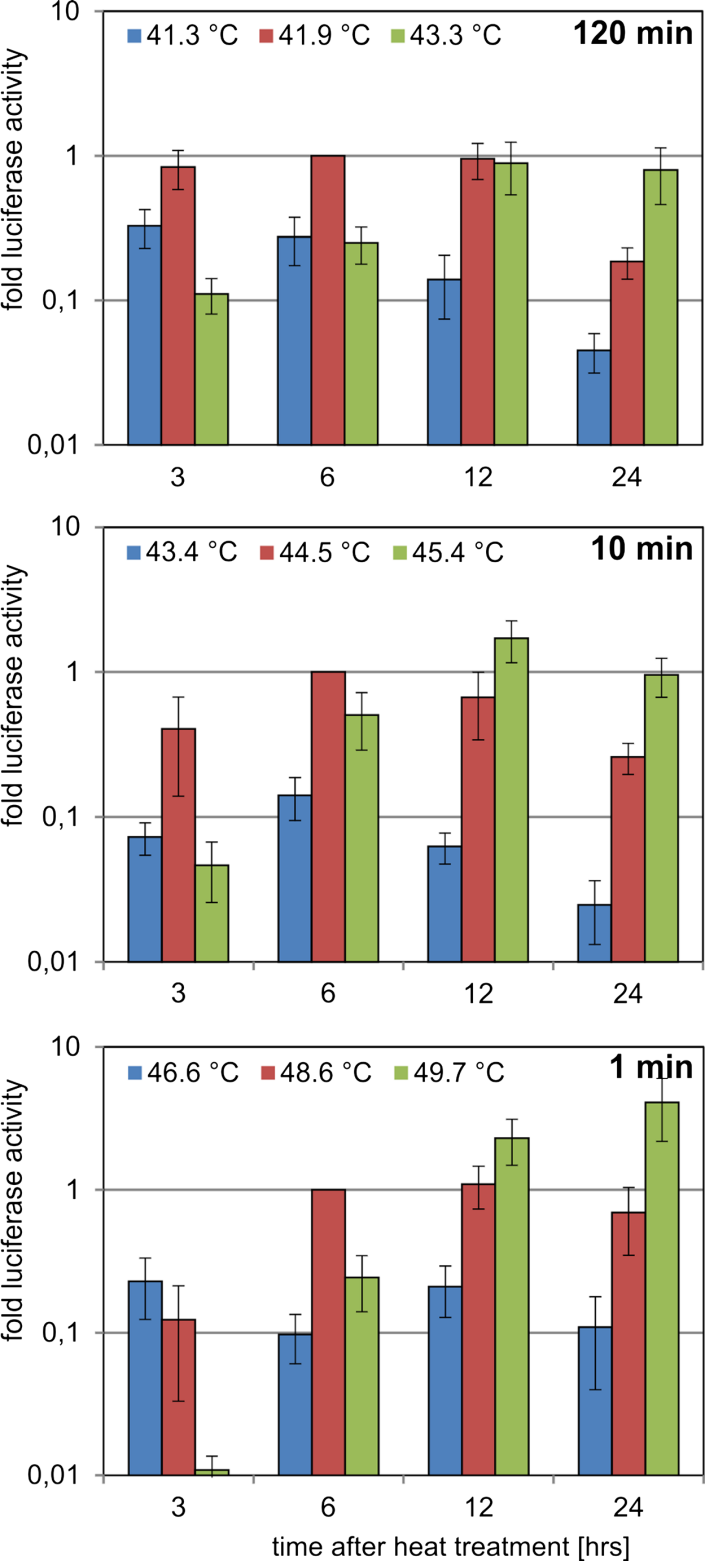
Luciferase activity of the HSE reporter for different recovery times after heat treatment. The HSE stable cell line was incubated for 120, 10 and 1 min at the indicated temperatures in a thermocycler. Luciferase activity was determined at the indicated recovery times after heat treatment and was normalised to the values for 6 h recovery time (41.9 °C for 120 min, 44.5 °C for 10 min and 48.6 °C for 1 min). All experiments were performed in triplicates, and SEM was calculated

The experiments so far were made with a fixed recovery time of 6 h for the cells after heat treatment. We previously detected maximum luciferase activity for the HSE promoter at this time point after 60 or 120 min exposure to 43 °C (Ortner et al. 2012). It could, however, be that shorter exposure times result in different kinetics. We therefore used 1, 10 and 120 min heat treatment and tested different recovery times (Fig. 5). The selected temperatures covered the spectrum of luciferase activity shown in Fig. 3 (one temperature at the peak luciferase level, one lower and one higher). In order to allow a better comparison, all values were normalised to the luciferase activity at 6 h (peak temperature) of each heat duration. As observed before (Fig. 3), all heat durations gave similar results at the respective temperatures. Interestingly, a clear trend was detectable for long recovery times, where the highest temperatures consistently resulted in stronger luciferase activity compared with lower temperatures. This trend was observed for all three exposure times but became most obvious for 1-min heat exposure, where the luciferase activity of high temperatures and late time points was higher than for the other conditions. Therefore, the peak levels of HS pathway activity are indeed shifted with altered recovery time, but interestingly these changes in kinetics are similar for both short and long heat treatment.

### Kinetics of natural versus artificial HS promoters

**Fig. 6:**
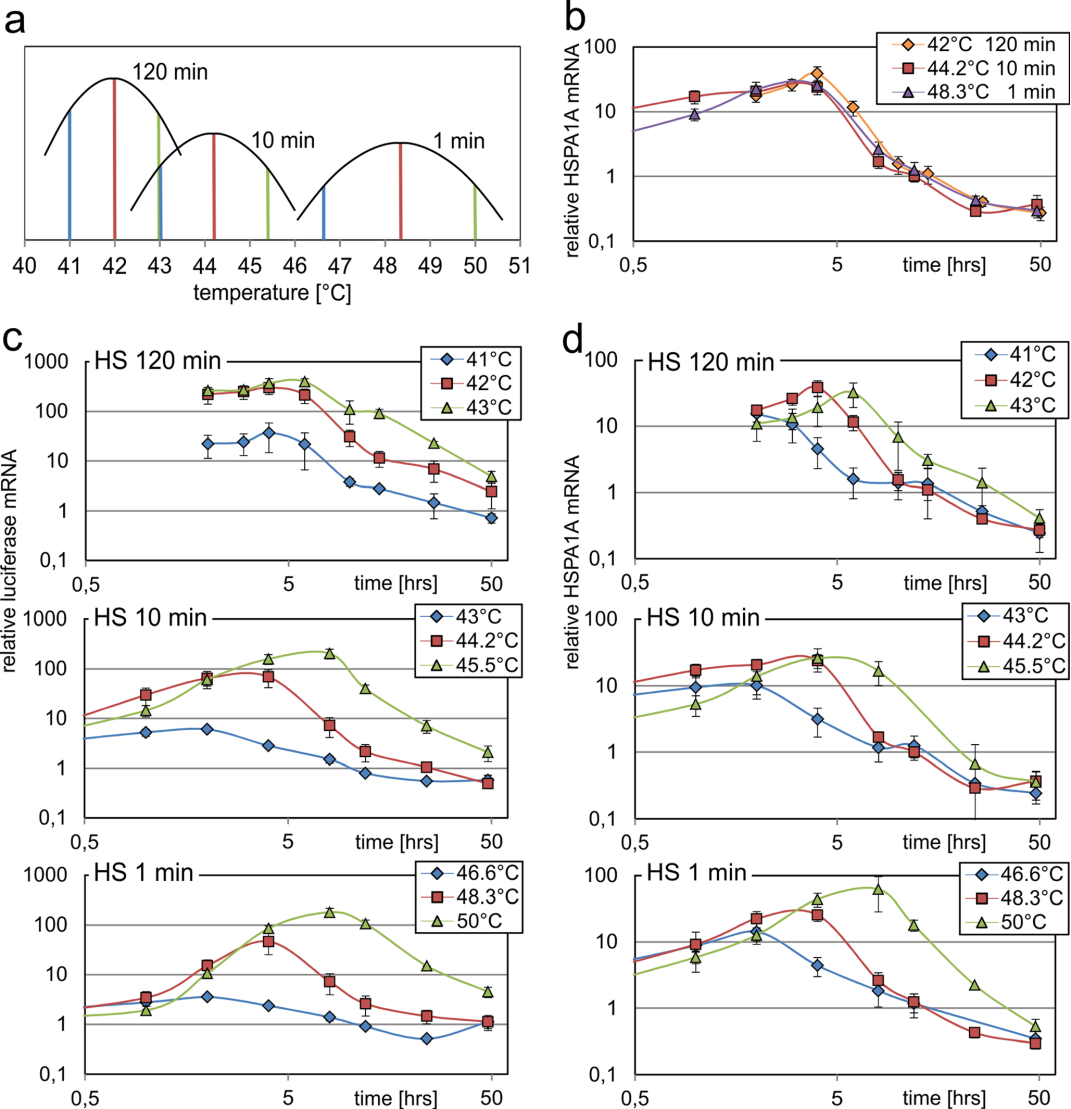
Comparing luciferase and HSPA1A mRNA levels for different stress conditions. The HSE stable cell line was incubated for 1, 10 or 120 at 41.0, 42.0 or 43.0 °C (120 min), 43.0, 44.2 or 45.5 °C (10 min) or 46.6, 48.3 or 49.9 °C (1 min) in a thermocycler and luciferase mRNA levels (**c**), and endogenous HSPA1A mRNA levels (**b**, **d**) were determined by a RT-qPCR up to 48 h after initiation of the heat treatment (the selection of temperatures is indicated in (**a**) using a schematic of the diagram shown in Fig. 3). The mRNA levels were normalised to that of cells incubated at 37 °C, and GAPDH was used as an internal reference. The *x*-axis in (**b**)–(**d**) represents the time after initiation of heat treatment. Note that the first measuring point for the 120-min exposure is therefore 2 h; the first time point for 10 min HS was 15 min and the first one for 1 min HS was 6 min, both are not shown in the diagrams. Time points were 1, 2, 4, 8, 12, 24 and 48 h. All experiments were performed in triplicates and SEM was calculated. For better comparison, the HSPA1A mRNA levels for 120 min at 42 °C, for 10 min at 44.2 °C and for 1 min at 48.3 °C are shown both in (**b**) and (**d**)

The HSE reporter provides clear advantages over natural HSP promoters for detection of HS pathway activity. Nevertheless, the majority of literature data on the HS response are based on HSP target gene activation, so we extended the comparison to the *HSPA1A* gene. But contrary to the previous experiments with promoter fragments, this time the regulatory regions in their natural chromatin context were analysed by RT-qPCR of endogenous mRNA. This furthermore allowed us to obtain translation independent data, since many stress-dependent regulatory mechanisms have been shown to act at the level of translation (Spriggs et al. 2010) and could thus affect the measured luciferase protein levels. As in the experiment before, we used different heat stress conditions and time points of recovery. The HSE stable cell line allowed us to directly compare luciferase mRNA levels originating from the HSE promoter with the mRNA levels of the endogenous HSPA1A gene within the same cells. As an internal reference, we used the house-keeping gene GAPDH, which is not affected by the heat treatment (Fig. S2). First, we performed time course experiments for 1, 10 and 120 min heat exposure at the temperatures resulting in peak promoter activity for the HSE promoter (48.3, 44.2 and 42.1 °C respectively for the three exposure times; red bars in Fig. 6a). Almost identical curves for the three different exposure times appeared when the HSPA1A mRNA levels were plotted against the time after heat treatment initiation (Fig. 6b, note that the 120-min curve therefore starts at 2 h). Peak levels of mRNA were detected at 4 h, which fits well to the maximum luciferase activity levels detected at 6 h (Fig. 5). Therefore, the kinetics of HSPA1A promoter activity is almost identical for long heat exposure at lower temperatures compared with short exposure times at high temperatures.

In addition to the peak activation temperatures for the different exposure times (120, 10 and 1 min), we next selected one temperature below and one above the peak position (Fig. 6a). Using three temperatures for each exposure time, we quantified the luciferase mRNA levels produced by the artificial HSE promoter over the time and compared it with the mRNA levels of the endogenous *HSPA1A* gene (Fig. 6c, d). In general, the mRNA levels were higher for the artificial promoter (note the different scale of the *y*-axis for the two promoters in Fig. 5c, d). This corresponds well to the induction rates of the two promoters observed in the transient experiments (Fig. 2c). In addition, the HSPA1A mRNA showed a tendency to earlier peak positions and subsequently faster reduction to basal levels, indicating a shorter half-life of the HSP mRNA compared with that of luciferase. Otherwise, the overall picture of the kinetics was quite similar for the two promoters. As seen before for the luciferase activity measurements (Fig. 5), the kinetics of the mRNA levels strongly differed between lower and higher temperatures for the same heat durations. Also at the level of mRNA, higher temperatures consistently resulted in later peak levels. This was not only observed for the HSE promoter but likewise occurred for the endogenous *HSPA1A* gene. In addition, these shifts were observed for all three heat durations in a similar manner. Therefore also at the level of kinetics, long and short heat durations result in highly similar HS pathway activation, although at completely different temperatures.

## Discussion

HSPs are chaperones which fulfil critical functions in proteostasis, in normal but in particular also in stressed cells (Richter et al. 2010). Their activity is therefore regulated by multiple stress-dependent and stress-independent pathways, resulting in highly specific expression characteristics within different tissues. HSPA1A is one of the strongest induced genes upon activation of the HSF-mediated HS response (Trinklein and Murray 2004). It has therefore repeatedly been used as a marker gene for HS pathway activity. However, it is also affected by multiple other pathways like the hypoxia pathway (Gogate et al. 2012), Keap1-Nrf2-ARE pathway (Hensen et al. 2013), MAPK/AP1 pathway (Mendillo et al. 2012), TGF-ß pathway (Takenaka and Hightower 1992), NF-B and CREB-mediated signalling (Sasi et al. 2014). As a consequence, the *HSPA1A* gene shows high basal expression levels and tissue-specific activity (Huang et al. 2001); Trautinger et al. 1993). Heat stress strongly activates the HS response; however, other stress pathways are also affected. Depending on the various stress conditions, a network of different stress pathways is therefore activated and the HSP target gene promoters integrate these signals in a highly specific manner. On the level of target gene transcription, a separation of these stress pathway activities is therefore extremely difficult. But contrary to other stress pathways, the HS response is mediated by HSF1 activation. An ideal reporter for this pathway should therefore exclusively react to this transcription factor. We made a promoter existing only of HSEs. It shows extreme low background levels in all cell lines we tested, indicating no crosstalk by other pathways. On the contrary, the HSPA1A promoter under the same conditions showed high basal levels, indicating that the abovementioned pathways are partially active at the uninduced state. Due to the multimerisation of the consensus HSEs, the artificial HSE promoter is highly inducible. We repeatedly measured several thousand fold increase of luciferase activity upon heat treatment. Although the *HSPA1A* gene is one of the best induced HSF targets, it does not reach the induction levels of the artificial construct.

The superior induction of the artificial reporter constructs could be verified in several cell lines (Fig. S1; with the single exception of HEK 293 T cells). Beside a 700-bp fragment, we also tested extended versions of the HSPA1A promoter, but none of these showed higher activities. Finally, we also compared HSE promoter activity to transcription from the endogenous regulatory regions of the *HSPA1A* gene (Fig. 6), thereby excluding the possibility that additional not yet recognised elements outside the promoter enhance the stress-dependent transcription of the gene within its natural chromatin environment. However, the induction rates of the mRNA originating from the endogenous gene also did not reach those of the HSE promoter integrated into the genome. The superior induction of the artificial construct can be explained by the presence of only three HSEs in the natural HSPA1A promoter (Fig. 1). In the artificial promoter, an increase from three to five HSEs led to a clear enhancement of transcriptional activity (Fig. 2b). Obviously, a selection for more HSEs and consequently higher induction rates did not take place during evolution of the HSP promoters, but a high degree of multimerisation strongly increases the sensitivity of the artificial HSE reporter construct.

HSF1 is considered the key activator of the HS response (Anckar and Sistonen 2011). The HSE promoter should therefore be strongly dependent on this factor. HSF2 has similar DNA-binding properties as HSF1 and in certain conditions more strongly binds to genomic targets (Vihervaara et al. 2013). The transcriptional activity of HSF2 is weak, but nevertheless can affect expression of certain target genes considerably (Ostling et al. 2007). Therefore in addition to HSF1, HSF2 would also be a candidate for activation of the HSE promoter. Due to the simple design of the promoter, non-HSF activities are unlikely to activate the promoter and indeed, the HSE promoter showed extreme low basal activities in all cell lines tested. Nevertheless, a key experiment was the behaviour of the reporter in absence of HSF1. We tested this in HSF1 (−/−) MEF cells, which express HSF2 (McMillan et al. 2002; Ostling et al. 2007) and found a very low luciferase activation of ~4-fold upon heat exposure, whereas the same treatment resulted in several thousand-fold activation of the reporter in wildtype MEF cells. Therefore, HSE reporter activation strictly depends on the presence of HSF1.

The above-discussed dependency of HSP gene promoters on multiple pathways and tissue specific transcription factors explains the strong deviations in HSP expression reported for different cell types (Akerfelt et al. 2010). Surprisingly, we saw little variations of HS response activity in experiments performed with the HSE promoter. In all cases, we observed substantial pathway activity at similar temperatures (Fig. S1), except for the HSF1 (−/−) cells. The absolute numbers of induction varied, however these numbers strongly depend on measurements in 37 °C reference cells, where the extreme low levels of luciferase activity are affected by multiple experimental parameters and are difficult to compare between cell lines. Nevertheless, our experiments suggest a highly reproducible HS pathway activity in most cells, contrary to expression levels of the HSP target genes affected by multiple pathways.

In addition to transcriptional initiation, other mechanisms have been proposed to regulate HSPA1A expression during stress conditions. Transcriptional pausing was first described for the *Drosophila HSP70* gene and was thought to be specifically involved in stress-induced expression (O’Brien and Lis 1991). In general, the blocking of RNA polymerase elongation depends on sequence elements in the 5′ region of the gene (Levine 2011). Similar mechanisms have been proposed for mammalian HSP genes; in addition, specific chromatin arrangements were suggested to regulate stress-dependent transcription (Brown et al. 1996). The direct comparison of our artificial promoter with an endogenous HSP gene allowed us to evaluate the influence of these mechanisms on heat stress-dependent expression. The artificial promoter represents a minimal version of a HS-responsive regulatory region. It consists exclusively of multimerized HSEs, and therefore lacks elements affecting transcriptional pausing and specific nucleosome positioning. However, it showed high transcriptional inducibility in human cells, but it was not clear whether the minimal arrangement of HSEs in the artificial promoter could mimic the properties of a complicated regulatory unit embedded in its optimised genomic context. Nevertheless, the HSE promoter showed stronger activity, both transiently and after stable integration into the genome (almost 10-fold higher mRNA induction), but otherwise behaved highly similar to the HSPA1A regulatory regions. The mRNA stability was lower for the natural promoter, which is typical for a strongly induced gene and allows rapid recovery to basal levels after the stress conditions. We cannot exclude that transcriptional pausing or specific nucleosomal arrangements favour a faster onset of transcription, but the overall picture of the expression kinetics was highly similar for both regulatory regions at the same temperatures and heat durations. Therefore isolated HSEs can mimic the expression characteristics of a complex HSP regulatory region in response to heat stress, regulation at the chromatin level and transcriptional pausing seem to play minor roles in HS-dependant expression in mammalian cells, contrary to *Drosophila* (Amin et al. 1985). In the meantime, genome-wide exploration has uncovered that a large fraction of all genes are regulated by transcriptional pausing, most of them independently of stress conditions (Levine 2011).

Heat exposure during burn injuries typically lasts for seconds. Thermal transfer in the tissue slows down the process, but nevertheless the heat exposure times often do not extend 1 min. On the other hand the temperatures in the tissue can reach 60 °C (Shupp et al. 2010), which is far beyond the survival limit of cells in conventional HS experiments but might be explained by the short duration time. We hypothesised that under these conditions, not only the survival temperature is shifted to higher temperatures but also the HS response of the cells. We, therefore, compared long exposure times to short pulses of heat.

HS pathway activation was previously shown to occur at high temperatures and short heat exposure (Kim et al. 1995); (Fujitomi et al. 1999); (O’Connell-Rodwell et al. 2004); (Kruse et al. 2008). Here, we show that the HS response strictly depends on a combination of the temperature and the exposure time (Fig. 3). Protein denaturation is considered as the main trigger mechanism for HS pathway activation (Morimoto 1998) and is known to depend on temperature, as well as on the time of heat exposure (Weijers et al. 2003). Our data perfectly fit to such a model. On the contrary, thermosensor based models postulate a threshold temperature as trigger mechanism. Indeed, such mechanisms have been described for bacteria and plants (Horváth et al. 2012). In mammals, the cytoplasmic membrane (Török et al. 2013) and the RNA-based cofactor HSR1 (Shamovsky and Nudler 2008) were proposed to mediate such temperature triggered mechanisms; however, our data do not support this view. Even between 60 and 120 min exposure time, clear differences in the peak temperature are detectable and argue against a threshold-based mechanism. Therefore, a combination of exposure time and temperature determines HS pathway activation also for short exposure times as occurring in burn injuries.

Independent of the exposure times, we saw differences in the kinetics of the HS response. In all cases, higher temperatures resulted in delayed transcription, whereas lower temperatures led to faster activation. We obtained the same results for WI-38 human fibroblast cells (data not shown); similar observations were also made with laser exposures of transgenic animals (Mackanos and Contag 2011). The shift of high temperatures to later peak activities was particularly evident for short exposure times. Early recovery times at these conditions resulted in low activities, whereas this was strongly compensated at late time points (highest activities for both protein and mRNA levels, compare Figs. 5 and 6c, d). This delayed HS response might have important therapeutic implications for patients with burn injuries, since the lag particularly affects those cells which were exposed to the highest temperatures and consequently would need immediate onset of this critical emergency pathway. Treatment of the burn wounds with HS inducers could represent a potential strategy to improve cell survival, which is of key importance for wound healing (Orgill et al. 2005). Potential candidate molecules for pharmacologic HS induction have also been discussed for treatment of neurodegenerate diseases (Neef et al. 2011).

Taken together, we established a highly sensitive and selective reporter for HS pathway activity. Due to multimerisation, the HSEs of the promoter are strongly activated by cellular stress. Based on the fact that the promoter contains no other elements than HSEs, it exclusively reacts to the HS pathway and shows extreme low basal activity in non-stressed cells. The reporter strictly depends on the presence of HSF1 and is therefore ideal for selective detection of HSF1 mediated HS pathway activity. The artificial HSE reporter revealed similar expression characteristics for the HS response when extended heat durations with typical HS temperatures of 41 to 43 °C were compared with temperatures up to 50 °C and exposures of 1 min. On the other hand, different temperatures for the same heat durations resulted in considerable differences in the kinetics of the response.

## Electronic supplementary material

Below is the link to the electronic supplementary material.

Fig. S1 HS pathway activity in different cell lines. The HSE and the HSPA1A promoter were transiently transfected into the indicated cell lines. Heat treatment was performed for 1 h at the indicated temperatures and luciferase activity measured after 6 h. One representative experiment is shown for each cell line. Note that only in HEK 293 T cells the HSPA1A promoter showed higher luciferase activity compared with the HSE promoter. (PDF 44 kb)

Fig. S2 GAPDH mRNA levels are not affected by heat treatment. HEK 293 cells were incubated for 120 min at 42 °C and lysed at the indicated time points. Mean values of GAPDH mRNA levels measured in three independent experiments are shown relative to those for 37 °C reference cells (*blue*). Contrary to the results shown in Fig. 6, these values are not normalised to an internal reference and therefore show fluctuations depending on the conditions of cDNA preparation; however, no tendency for heat dependent regulation is seen compared with HSPA1A levels measured in the same cells (*red*). (PDF 14 kb)
