# Supplementary figures and images for: An artificial HSE promoter for efficient and selective detection of heat shock pathway activity
Source: Cell Stress Chaperones. 2014 Aug 29;20(2):277–88. doi: 10.1007/s12192-014-0540-5 (PMC4326385; doi:10.1007/s12192-014-0540-5)

**HeLa**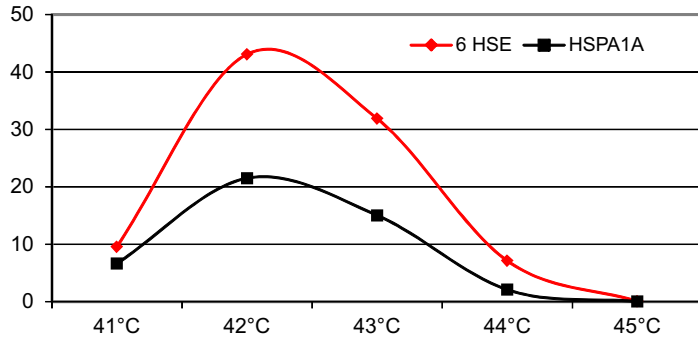**HaCaT**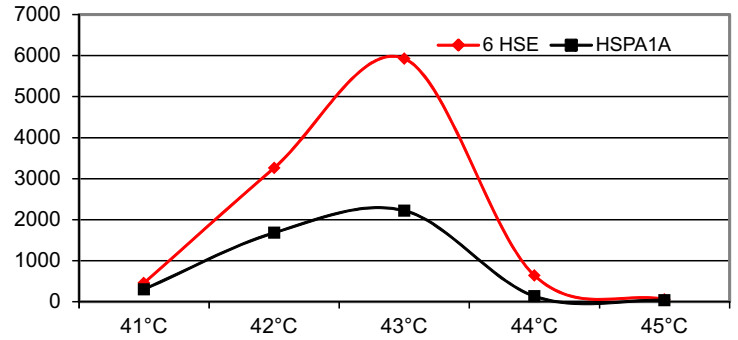**MCF-7**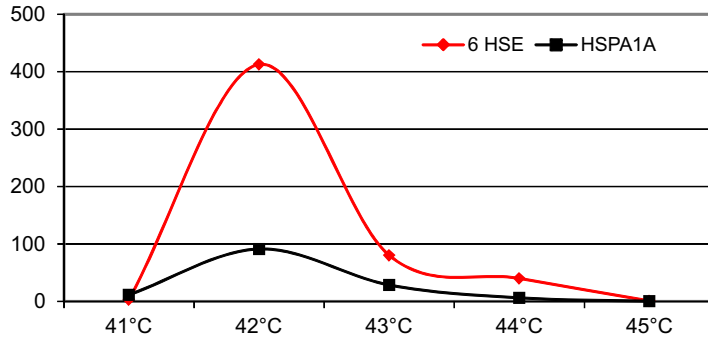**NIC NIH-383**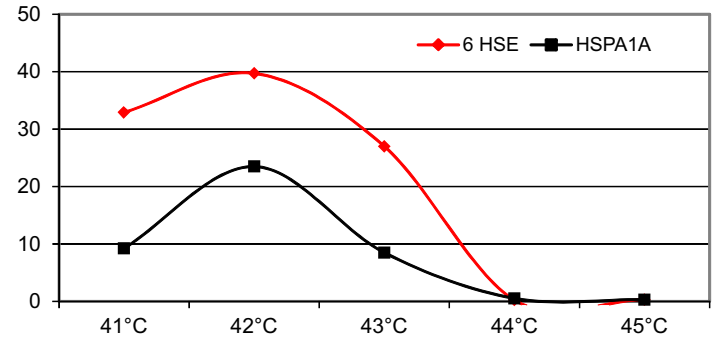**SK-BR3**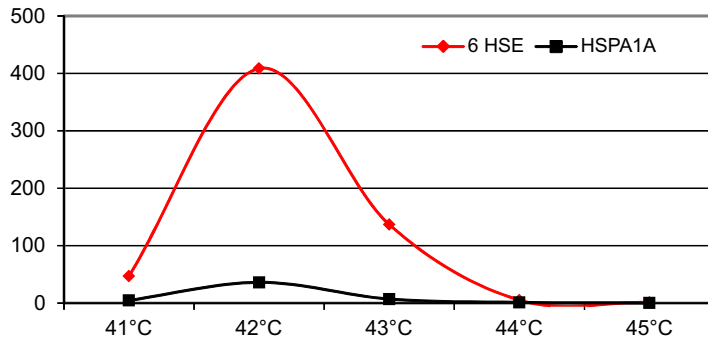**HEK 293T**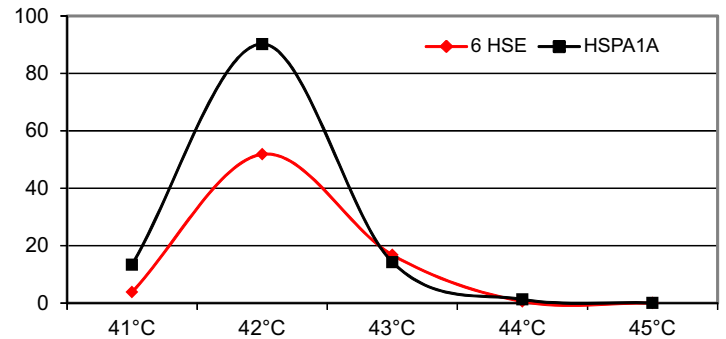

Supplement: Supplementary file 1 — HS pathway activity in different cell lines. The HSE and the HSPA1A promoter were transiently transfected into the indicated cell lines. Heat treatment was performed for 1 h at the indicated temperatures and luciferase activity measured after 6 h. One representative experiment is shown for each cell line. Note that only in HEK 293 T cells the HSPA1A promoter showed higher luciferase activity compared with the HSE promoter. (PDF 44 kb) [file 12192_2014_540_MOESM1_ESM.pdf]

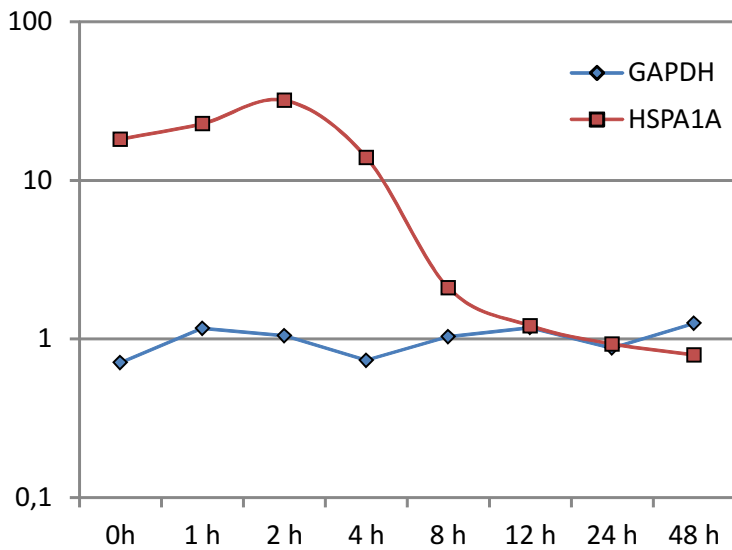

Supplement: Supplementary file 2 — GAPDH mRNA levels are not affected by heat treatment. HEK 293 cells were incubated for 120 min at 42 °C and lysed at the indicated time points. Mean values of GAPDH mRNA levels measured in three independent experiments are shown relative to those for 37 °C reference cells (blue). Contrary to the results shown in Fig. 6, these values are not normalised to an internal reference and therefore show fluctuations depending on the conditions of cDNA preparation; however, no tendency for heat dependent regulation is seen compared with HSPA1A levels measured in the same cells (red). (PDF 14 kb) [file 12192_2014_540_MOESM2_ESM.pdf]
